# Analysis of β-N-methylamino-L-alanine (BMAA) in spirulina-containing supplements by liquid chromatography-tandem mass spectrometry

**DOI:** 10.1186/2046-9063-10-5

**Published:** 2014-08-08

**Authors:** Pearse McCarron, Alan C Logan, Sabrina D Giddings, Michael A Quilliam

**Affiliations:** 1 National Research Council Canada, Measurement Science and Standards, Biotoxin Metrology, 1411 Oxford St, Halifax, NS B3H 3Z1, Canada; 2 CAMNR, 23679 Calabasas Road Suite 542, Calabasas, CA 91302, USA

**Keywords:** Spirulina, Dietary supplements, BMAA, β-N-methylamino-L-alanine, Food chain

## Abstract

Over the last decade the amino acid beta-*N*-methylamino-L-alanine (BMAA) has come under intense scrutiny. International laboratory and epidemiological research continues to support the hypothesis that environmental exposure to BMAA (e.g., through dietary practices, water supply) can promote the risk of various neurodegenerative diseases. A wide variety of cyanobacteria spp. have previously been reported to produce BMAA, with production levels dependent upon species, strain and environmental conditions. Since spirulina (*Arthrospira* spp*.*) is a member of the cyanobacteria phylum frequently consumed via dietary supplements, the presence of BMAA in such products may have public health implications. In the current work, we have analyzed ten spirulina-containing samples for the presence of BMAA; six pure spirulina samples from two separate raw materials suppliers, and four commercially-available multi-ingredient products containing 1.45 g of spirulina per 8.5 g serving. Because of controversy surrounding the measurement of BMAA, we have used two complementary liquid chromatography-tandem mass spectrometry (LC-MS/MS) methods: one based on reversed phase LC (RPLC) with derivatization and the other based on hydrophilic interaction LC (HILIC). Potential matrix effects were corrected for by internal standardization using a stable isotope labeled BMAA standard. BMAA was not detected at low limits of detection (80 ng/g dry weight) in any of these product samples. Although these results are reassuring, BMAA analyses should be conducted on a wider sample selection and, perhaps, as part of ongoing spirulina production quality control testing and specifications.

## Background

Over the last decade, an increasingly robust body of bench and epidemiological research has suggested that β-*N*-methylamino-L-alanine (BMAA) may play a role in various neurodegenerative diseases [1,2]. BMAA is a non-proteinogenic amino acid first identified in the seeds of *Cycas micronesia* in 1967 [3]. It was noted in the 1980s [4] and revisited again in 2002 [5], that cycad pulp and seed flour (as well as animals that fed upon cycad seeds) have been a significant part of the traditional dietary and medicinal agents among specific Western Pacific-dwelling communities (e.g. Guam). Based on experimental evidence of BMAA-induced neurotoxicity, these research groups theorized that a high incidence of neurodegenerative diseases in these regions may be associated with dietary intake of BMAA [4,5]. Investigations would confirm that BMAA is found in various dietary items along the regional food chain within these communities and that BMAA can accumulate in animal tissue over time [6].

Initially thought to be a relatively isolated concern traceable to the *Cycas*, and its seeds in particular, BMAA was reported in the brain tissue of a small sample of Canadian adults who had died in association with Alzheimer’s disease [7]. BMAA was not found in controls that had died from causes other than neurodegenerative. This suggested that BMAA may not merely be a local concern for those in Micronesia. A 2003 report indicated that BMAA was being produced by cyanobacteria that live in symbiotic fashion with the *Cycas* roots [8]. Subsequently, a study of 29 specific strains of free-living cyanobacteria (derived from marine, freshwater and brackish sources) showed that all but one produced BMAA [9]. Since the strains of cyanobacteria used in the study were drawn from diverse taxa, and given the ubiquity of the phylum cyanobacteria itself, the implications to public health could be significant.

Uncovering potential mechanisms of BMAA neurotoxicity is an area of intense research. Recent experimental studies indicate that BMAA induces oxidative stress and excitotoxicity, and the agent may be misincorporated into nerve cell proteins, causing neuronal damage [10,11]. These studies are matched by emerging epidemiological work suggesting that residential proximity to cyanobaterial-contaminated lakes is associated with increased risk of amyotrophic lateral sclerosis (ALS) [12,13]. Given the progress in this area, it is surprising that a significant source of direct cyanobacterial intake - spirulina-containing dietary supplements - have not been investigated for the presence or absence of BMAA.

Within the *Arthrospira* genus of cyanobacteria, *Arthrospira platensis* is a dietary supplement commonly referred to as ‘spirulina’. Although spirulina is sold as a stand-alone supplement, it is frequently incorporated into so-called ‘green drink’ supplements, those inclusive of a variety of fruit, vegetable, herbal and aquatically-derived extracts in powdered form. The dose of spirulina within these supplements is usually significant, typically ranging between 1–2 grams per serving. Given that such supplements are marketed for continuous use, the presence of BMAA would be cause for legitimate concern. Although commonly referred to as a “blue-green algae”, and remarkably still remains technically classified as a botanical under the existing rules of the International Code of Nomenclature of Prokaryotes, spirulina actually belongs to the phylum *Cyanobacteria* and lack the nuclei of algae [14].

We could find only a single published study, one conducted by Health Canada’s Bureau of Chemical Safety [15], which examined 11 “blue-green algae” containing food supplements for BMAA content. Using a liquid chromatographic method, the investigators found no BMAA to a detection limit of 200 ng/g. The researchers were able to recover up to 89% of BMAA when they intentionally spiked the supplements with BMAA, lending credibility to their findings. However, despite making note of the connection between BMAA and cyanobacterium spp. as a background to their investigation, within the methods section the group used only the broad term of “blue-green algae food supplements” and they did not specify species or provide a detailed description of the algae within the 11 products in question. It is unclear if the researchers were intending to examine spirulina, underscoring the need for proper nomenclature. Therefore, the presence/absence of BMAA in products specifically listing spirulina (or *Arthrospira spp.*) as an ingredient remains an open question.

Furthermore, advanced analytical techniques have since allowed for improved detection of BMAA [16-20], including methodology that allows for the consideration of reactivity between BMAA and metal ions [21]. Advanced techniques indicate that the broad assumption that all (or 90% plus) members of the cyanobacterial taxa are BMAA producers may be incorrect [22]. Indeed, the most extensive review of the topic to date [23] has suggested that many of the assumptions related to the presence of BMAA in aquatic biosystems are based on a background of inadequate analytical methods and false positives. This same review highlights that liquid chromatography-mass spectrometry (LC-MS/MS) is currently regarded as the most suitable method to confirm the presence of BMAA and measure its concentration in samples. Still, to the best of our knowledge, researchers have not specifically looked for BMAA in edible spirulina samples. Given the international popularity of spirulina as a dietary supplement [24,25], particularly in the context of “green drinks”, we have determined the BMAA content of ten spirulina samples using two LC-MS/MS methods. Our samples were comprised of six raw material samples of pure spirulina (derived from two separate suppliers) and four commercially available Canadian spirulina-containing dietary supplements.

## Methods

### Reagents

BMAA (β-*N*-methylamino-L-alanine hydrochloride, B107, 10 mg) was obtained from Tocris Bioscience (distributor: R&D systems, Minneapolis, MN), AEG (N-(2-aminoethyl)-glycine) was from TCI America chemicals (Portland, OR), and DAB (DL-2,4-diaminobutyric acid dihydrochloride, B3758, 1 g) was from Sigma Aldrich (Oakville, ON Canada). Ammonium formate (99%), sodium hydroxide, d3-methylamine hydrochloride, 2-acetamidoacrylic acid, and hydrochloric acid were purchased from Sigma Aldrich (Oakville, ON, Canada). Distilled-in-glass ethanol and methanol and HPLC grade acetonitrile were obtained from Caledon (Georgetown, ON, Canada) or BDH Inc. (Toronto, ON, Canada). Formic acid (>98% ACS grade) was obtained from EMD (Gibbstown, NJ, USA). The internal standard, d3-BMAA, was synthesized in-house following previously outlined procedures with some modifications [17,26,27]. In a round bottom flask a solution of d3-methyl amine hydrochloric acid (1 g in 4 mL of water) was cooled to 4°C. Sodium hydroxide (0.88 g) was added while stirring and the reaction was allowed to warm to room temperature. Then 2-acetamidoacrylic acid (0.37 g) was added and stirred without heat for 10 min. On a heating mantle the reaction temperature was brought to 35°C for 22 hr. After cooling to room temperature the mixture was dried using a rotary evaporator and the crude product was dissolved in a minimum volume of 3 M HCl and refluxed for 2 hr. The HCl was removed and the residue recrystallized from ethanol and water at 4°C. The resulting crystals were collected by filtration, washed with cold ethanol and dried. The structure of the product d3-BMAA was verified by NMR.

### Samples and preparation

Spirulina samples, ten in total, included six samples of raw spirulina furnished from two separate spirulina manufacturers, and four samples of multi-ingredient “green drink” products that were inclusive of 1.45 g of spirulina per serving. All samples were forwarded with commercial labels removed and coded as #1-10, i.e., all samples arrived to the NRC office as blind samples. The samples were stored at room temperature until analysis by LC-MS/MS. A positive control Cycad plant (*Cycas debaoensis*) was obtained from Jurassic Plants Nursery (Halfmoon Bay, BC). Leaves of the cycad plant were chopped into fine pieces using a scalpel and then freeze-dried in preparation for extraction and analysis.

Bulk samples were thoroughly mixed and 10 mg aliquots were transferred to glass extraction tubes. A 20 μL aliquot of d3-BMAA internal standard was added to each sample and then 1 mL of 6 mM HCl was added and vortex mixed. The tubes were purged with nitrogen, covered with Teflon tape, capped and maintained at 110°C for 18 hr in order to determine “total BMAA”. When cool, the hydrolyzed sample extracts were filtered (0.22 μm Ultrafree-MC) and then dried under nitrogen at 55°C and reconstituted in 1.0 mL of 2 mM HCl. For RPLC the extracts were derivatized as described below. For HILIC a 100 μL aliquot of each filtered sample was dried under nitrogen and reconstituted in 100 μL of acetonitrile/water (65:35).

### AQC derivatization

AccQ-Tag kit reagent (Waters, Milford, MA, USA) was prepared according to the manufacturer’s instructions. For AQC derivatization, 40 μL of sample extract (dissolved in 2 mM HCl) was placed in a clean LC vial and dried thoroughly under a nitrogen stream at 55°C. The residue was reconstituted in 120 μL of AccQfluor borate buffer solution and vortex mixed. A 40 μL aliquot of AQC reagent solution was added and vortex mixed. The solution was allowed to sit at room temperature for 1 min and then heated at 55°C for 10 min before transfer to an LC insert vial for LC-MS/MS.

### LC-MS/MS

Quantitative measurement of BMAA, DAB and AEG as AQC derivatives was performed using RPLC-MS/MS on an Agilent 1200 LC system (Agilent Inc., Palo Alta, CA) connected to an API4000 QTRAP mass spectrometer (AB Sciex, Concord, ON, Canada) using electrospray ionization and selected reaction monitoring. Direct quantitation of BMAA, DAB and AEG by HILIC-MS/MS on an Agilent 1100 LC system connected to an API 4000 mass spectrometer. Table 1 provides the experimental details for the two methods.

**Table 1:** Experimental conditions for LC-MS/MS analyses

	**Method 1: AQC-RPLC-MS/MS**						**Method 2: HILIC-MS/MS**					
Derivatization	AQC						none					
Chromatography	RPLC						HILIC					
Stationary phase	Thermo Hypersil Gold C18						TosohBioscience TSK-gel Amide-80					
Column length × I.D. (mm)	50 × 2						250 × 2					
Column packing size (μm)	1.9						5.0					
Column temp (°C)	20						40					
Mobile phase A	Water with 20 mM NH_4_COOH (pH 5 adjusted)						Water with 50 mM HCOOH					
Mobile phase B	Methanol						Acetonitrile: Water (95:5) with 50 mM HCOOH					
Flow rate (mL/min)	0.40						0.20					
Injection volume (uL)	6.0						5.0					
Elution conditions	10-40% B in 6 min, to 85% B at 6.1 min, hold at 85% B until 8.5 min.						90 to 60% B in 15 min, hold to 20 min, decrease to 55% B at 21 min, hold to 30 min.					
MS Ionization	Positive electrospray (Turbospray®)						Positive electrospray (Turbospray®)					
MS source temp (°C)	450						450					
MS ionization voltage (V)	5500						5500					
MS orifice potential (V)	70						40					
				**Relative intensities**						**Relative intensities**		
**SRM transitions for:**	**Precursor m/z**	**Product m/z**	**CE (V)**	**BMAA**	**DAB**	**AEG**	**Precursor m/z**	**Product m/z**	**CE (V)**	**BMAA**	**DAB**	**AEG**
1 BMAA, DAB, AEG	459.2	171.1	35	1.00	1.00	1.00	119.1	102.1	15	1.00	0.40	1.00
2 BMAA, DAB, AEG	459.2	119.1	30	0.28	0.07	0.28	119.1	101.1	20	0.55	1.00	0.65
3 BMAA, DAB, AEG	459.2	289.1	20	0.39	0.13	0.27	-	-	-	-	-	-
4 BMAA specific	459.2	258.1	30	0.04	na	na	119.1	88.1	20	0.29	na	na
5 DAB specific	459.2	188.1	35	na	0.02	na	119.1	74.1	25	na	0.11	na
6 AEG specific	459.2	214.1	35	na	na	0.02	-	-	-	-	-	-
7 d3-BMAA	462.2	292.1	20	1.0	na	na	122.1	105.1	15	1.00	na	na
8 d3-BMAA	462.2	122.1	30	0.7	na	na	122.1	88.1	20	0.24	na	na
8 mono-AQC BMAA	230.1	171.1	30	na	na	na	na	na	na	na	na	na
9 di-AQC lysine	487.2	171.1	25	na	na	na	na	na	na	na	na	na
10 mono-AQC lysine	317.1	171.1	30	na	na	na	na	na	na	na	na	na

## Results and discussion

**Figure 1:**
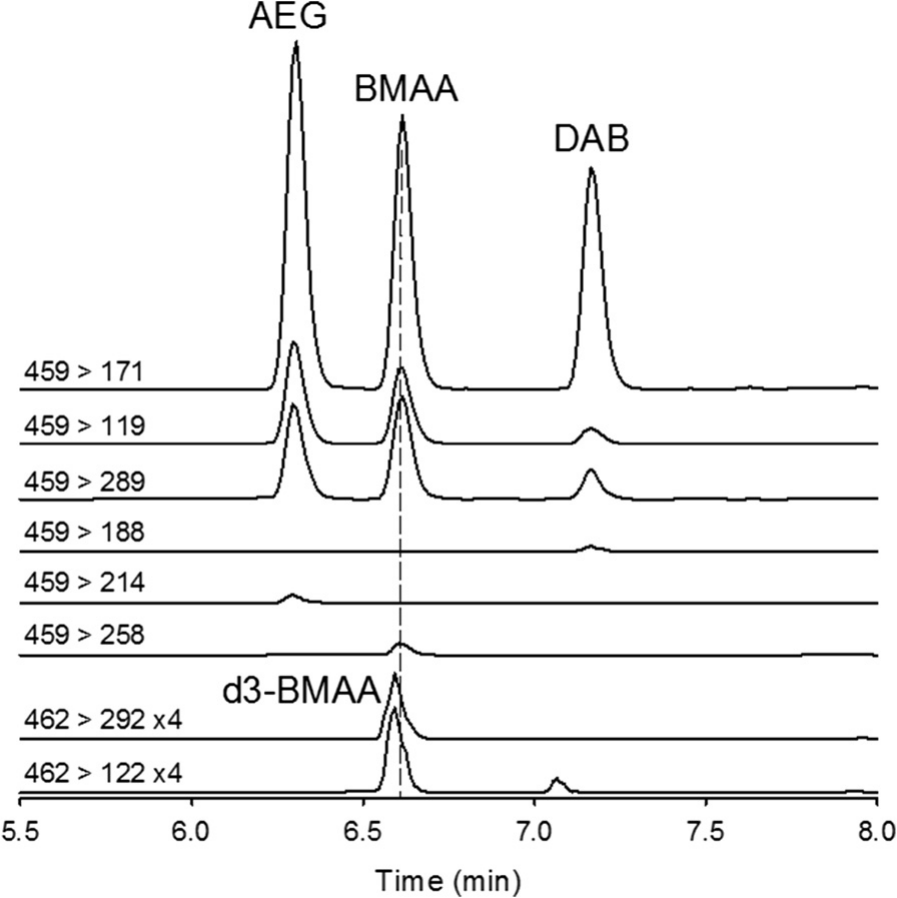
Chromatograms from AQC-RPLC-MS/MS analysis of standards.

Two complementary LC-MS/MS methods were used in the present study for the analysis of BMAA in hydrolysed spirulina-containing ‘green drink’ supplements with an extraction procedure aimed at providing “total BMAA” concentration, i.e., free plus protein-bound. One method was based on reversed phase LC (RPLC) with derivatization using 6-aminoquinolyl-N-hydroxysuccinimidylcarbamate (AQC) [16-19] and the other used hydrophilic interaction LC (HILIC) without derivatization [18]. Two other compounds, 2,4-diaminobutyric acid (DAB) and *N*-(2-aminoethyl)-glycine (AEG), were also monitored since they are isomeric with BMAA and have been reported in cyanobacterial samples [19,20]. A stable-isotope labeled internal standard, d3-BMAA, was used as internal standard in both methods to correct for extraction recovery and matrix effects, the latter being commonly observed in electrospray LC-MS/MS. Although better detection limits could have been achieved with solid phase extraction [18], such cleanup of samples was not used in this study because of concerns expressed in the literature [21] that BMAA might be lost during such procedures.Significant attention was paid to ensuring good separation of BMAA from isomeric compounds. There are many possible isomers of BMAA but the primary ones of concern are AEG and DAB. Good separations of BMAA from AEG and DAB were achieved in both RPLC and HILIC methods. Retention times for BMAA, AEG and DAB were 6.62, 6.31 and 7.17 min for RPLC (see Figure 1) and 18.46, 20.59 and 19.60 min for HILIC (data not shown), respectively. Target analytes were all baseline resolved as shown in Figure 1. The d3-BMAA internal standard eluted slightly earlier by 0.02 min than the unlabeled BMAA in RPLC and slightly later by 0.06 min in HILIC, but it was deemed that the peaks were sufficiently co-eluting in both methods as required for correction of matrix effects.

The selected reaction monitoring (SRM) transitions used for detecting BMAA and its isomers compounds are detailed in the Methods section (Table 1). A total of 6 transitions were used for detecting BMAA, AEG and DAB, three of which were common to all compounds and three of which were selective to each compound (see Figure 1). Unfortunately, the compound specific signals were much weaker and are therefore only useful for higher levels. The ratios of the transition intensities as well as the retention times relative to d3-BMAA were used as criteria for identification of the compounds. Excellent linearity of response was observed for the two methods (R^2^ > 0.99).

The limits of detection (LODs) were measured using calibration standards and were defined as the level at which there was a signal to noise ratio of 3. The LODs for the AQC-RPLC-MS/MS method were 80, 36 and 211 ng/g (dry weight) for BMAA, AEG and DAB, respectively. The corresponding LODs for HILIC-MS/MS were 93, 44 and 205 ng/g (dry weight). The absence of a signal for BMAA along with the presence of a signal for the d3-BMAA spike can be considered very good proof of absence (above the LOD) of BMAA in samples.

The suitability of the two methods, from sample extraction through to analysis, was tested with a positive control: an extract of leaves from a Cycad plant (*Cycas debaoensis*) (data not shown), which, as previously discussed, is known to support cyanobacterium spp. that produce BMAA. BMAA was detected and confirmed to be present at ~220 μg/g (dry weight).

**Figure 2:**
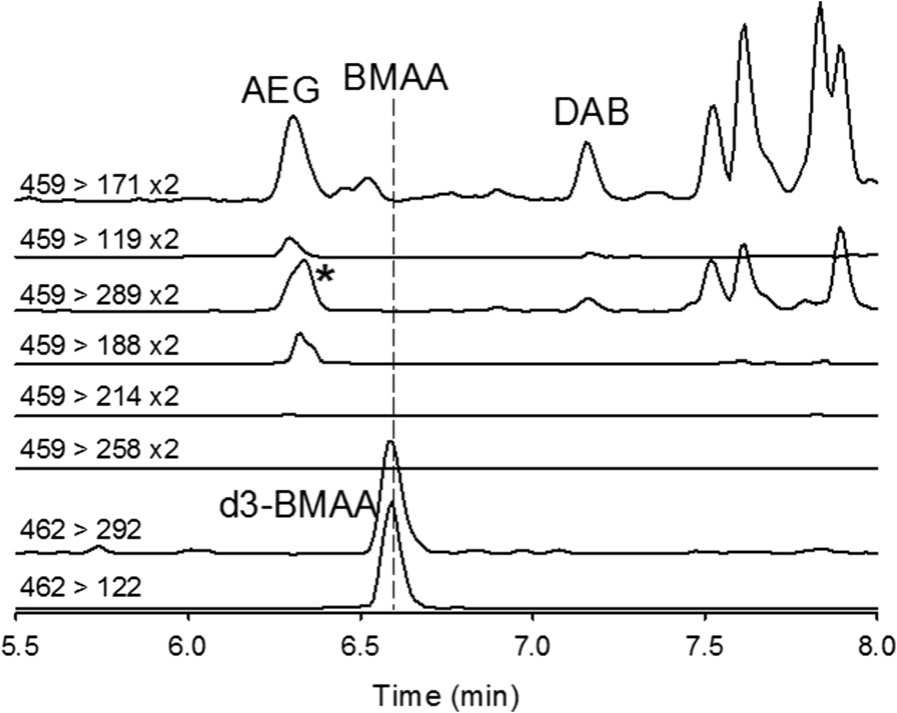
**Chromatograms from AQC-RPLC-MS/MS analysis of sample 7 spiked with 1** **μg/g d3-BMAA.**

Table 2 summarizes the results for the various samples analyzed. BMAA was not detected in any of the samples by either LC-MS/MS method. DAB and AEG were detected and confirmed in all samples at levels between 190 and 2600 ng/g (dry weight). Figure 2 shows a typical data set from the RPLC-MS/MS analysis of sample #7. No BMAA signal could be detected but signals for AEG, DAB and d3-BMAA were clearly observed at their correct retention times, which were very reproducible with less than 1% variation between samples. Measuring retention times relative to that of d3-BMAA allowed excellent matching (see Table 2). A number of peaks of unknown identity were observed at various retention times different than the target analytes, but are often only observed in one or two transition channels. This indicates the importance of using at least two, preferably three, SRM transitions for confirmation of compound identity. In this particular sample, a compound eluting close to AQC-derivatized AEG with a signal in the m/z 459 > 289 channel was observed at a low level (marked with an asterisk in Figure 2). We observed that this peak shifted its position relative to other peaks, sometimes approaching that of BMAA, when a new mobile phase batch with a slightly different pH was used. Analysis of the samples by the HILIC-MS/MS method were somewhat complicated by the effect of matrix on retention times, which shifted to earlier positions in the presence of matrix. Spikes of samples with BMAA confirmed, however, that d3-BMAA and BMAA still essentially co-eluted and the absence of a BMAA signal in samples could still be determined. This problem can be overcome with an SPE cleanup of the sample [18] and this will be used in future studies.

**Table 2:** Relative retention times (RRTs) and concentrations (ng/g) measured for AEG, BMAA and DAB in samples and in Cycad control material as analyzed by the RPLC-MS/MS method

	RRTs*			Concentrations (ng/g)**		
Sample #	AEG	BMAA	DAB	AEG	BMAA	DAB
GH-1	*nd*	*nd*	1.08	*nd*	*nd*	1226
GH-2	0.96	*nd*	1.09	290	*nd*	2493
GH-3	0.96	*nd*	1.09	191	*nd*	1773
GH-4	0.96	*nd*	1.09	569	*nd*	2569
GH-5	0.96	*nd*	1.09	1348	*nd*	874
GH-6	0.96	*nd*	1.09	1240	*nd*	1599
GH-7	0.96	*nd*	1.09	1136	*nd*	963
GH-8	0.96	*nd*	1.09	486	*nd*	490
GH-9	0.96	*nd*	1.09	290	*nd*	1270
GH-10	0.96	*nd*	1.09	500	*nd*	696
*Cycad (NRC)*	0.96	1.003	1.08	375	208,089	631

*relative to d3-BMAA (RT = 6.60 min).

**internal standard quantitation was used for BMAA, while external calibration.

*nd* = not detected (LOD = 80 ng/g for BMAA).

Retention times are expressed relative to a d3-BMAA internal standard that was spiked into the individual samples.

Recorded use of spirulina for medicinal and dietary use dates back to the 16th Century [14]. It has enjoyed a good degree of safety and a low risk of toxicity within the *in vitro* and *in vivo* models (although as mentioned, these have not addressed BMAA) [28]. Amidst concerns related to cyanobacteria spp. and neurodegenerative diseases, it is worth pointing out that spirulina has been the subject of a number of studies related to the brain and nervous system, with outcomes demonstrating benefit even in models of developmental origins of health and disease [29-33]. For example, oral spirulina administration has been shown to protect against experimentally-induced neural tube defects [34] and spirulina-enhanced diets afford neuroprotection in experimental models of Parkinson’s disease [35]. The anti-inflammatory and antioxidant properties of the agent in the context of neuroprotection have been well described [36].

In 2011, the Dietary Supplements Information Expert Committee of the United States Pharmacopeial Convention (USP) reviewed human and experimental studies, as well as regulatory and pharmacopeial sources before assigning *Spirulina maxima* and *Spirulina platensis* a Class A safety rating. Thus, these primary forms of spirulina, those most commonly found in supplements, became part of the National Formulary of the USP, a compendia recognized by the United States Food, Drug, and Cosmetic Act. However, the Expert Committee of the USP did acknowledge that the unknowns related to spirulina and BMAA were a valid concern and should be incorporated into manufacturing quality controls [14]. We agree. Indeed, although BMAA was not detected in any of the raw materials and finished product samples tested here, researchers have reported that BMAA production in various *Cyanobacteria* spp. is dependent upon nutrient availability and other environmental conditions [37]. Therefore, in an effort to ensure consumer safety and following the precautionary principle regarding the emerging BMAA research, routine testing using techniques such as those outlined in the current study should be given wider consideration by spirulina harvesters as part of standard quality controls.

## Conclusions

Previous research using older methodology has suggested that species from a wide variety of cyanobacteria phyla are capable of producing the neurotoxin BMAA. International studies indicate that BMAA may accumulate in tissue over time, suggesting that the regular consumption of dietary products containing BMAA is of concern to public health. Spirulina is a cyanobacterium widely consumed as a dietary supplement. Moreover, spirulina has emerged as an attractive and nutritious component of animal feed [38]; therefore the opportunity for biomagnification of BMAA into the food supply, should it be present in spirulina, is strong. Although spirulina has been found to have neuroprotective properties in various models, it had not, to the best of our knowledge, been subjected to research for BMAA determination – perhaps due to the positive findings on its neurological benefits.

Here, we have evaluated commercially available ‘green drink’ dietary supplements containing significant amounts of spirulina. Using two advanced LC-MS/MS techniques, we were unable to detect BMAA to a level of 80 ng/g in any of the product samples. Although reassuring, the small sample of pure spirulina and spirulina-containing retail products can only be considered as a preliminary step toward larger investigations and evaluation of quality control in spirulina production. In the often conflicting research surrounding the presence of BMAA in aquatic biosystems and products derived from fresh and marine waters, the use of LC-MS/MS is emerging as a gold standard with which to expand upon these discussions. The next steps should focus on inter-laboratory comparison studies and the production of certified reference materials for quality control of analyses.

## Competing interests

PM, SDG and MAQ report no conflicts of interest. ACL has received fees as an independent consultant for Genuine Health Inc, a manufacturer of dietary supplements containing spirulina.

## Authors’ contributions

PM, SDG and MAQ conducted all physical research related to sample testing; ACL coordinated the research project and all authors provided equal input in the manuscript preparation. All authors have read and approved the final manuscript.

## References

[B1] Bradley WG Borenstein AR Nelson LM Codd GA Rosen BH Stommel EW Cox PA Is exposure to cyanobacteria an environmental risk factor for amyotrophic lateral sclerosis and other neurodegenerative diseases? Amyotroph Lateral Scler Frontotemporal Degener 2013 14 325 333 23286757 10.3109/21678421.2012.750364

[B2] Chiu AS Gehringer MM Welch JH Neilan BA Does α-amino-β-methylaminopropionic acid (BMAA) play a role in neurodegeneration? Int J Environ Res Public Health 2011 8 3728 3746 22016712 10.3390/ijerph8093728 PMC3194113

[B3] Vega A Bell EA α-amino-β-methylaminopropionic acid, a new amino acid from seeds of *Cycas circinalis* Phytochem 1967 6 759 762

[B4] Spencer PS Nunn PB Hugon J Ludolph AC Ross SM Roy DN Robertson RC Guam amyotrophic lateral sclerosis-parkinsonism-dementia linked to a plant excitant neurotoxin Science 1987 237 517 522 3603037 10.1126/science.3603037

[B5] Cox PA Sacks OW Cycad neurotoxins, consumption of flying foxes, and ALS-PDC disease in Guam Neurology 2002 58 956 959 11914415 10.1212/wnl.58.6.956

[B6] Banack SA Cox PA Biomagnification of cycad neurotoxins in flying foxes: implications for ALS-PDC in Guam Neurology 2003 61 387 389 12913204 10.1212/01.wnl.0000078320.18564.9f

[B7] Murch SJ Cox PA Banack SA Steele JC Sacks OW Occurrence of beta-methylamino-l-alanine (BMAA) in ALS/PDC patients from Guam Acta Neurol Scand 2004 101 267 269 15355492 10.1111/j.1600-0404.2004.00320.x

[B8] Cox PA Banack SA Murch SJ Biomagnification of cyanobacterial neurotoxins and neurodegenerative disease among the Chamorro people of Guam Proc Natl Acad Sci U S A 2003 100 13380 13383 14612559 10.1073/pnas.2235808100 PMC263822

[B9] Cox PA Banack SA Murch SJ Rasmussen U Tien G Bidigare RR Metcalf JS Morrison LF Codd GA Bergman B Diverse taxa of cyanobacteria produce beta-N-methylamino-L-alanine, a neurotoxic amino acid Proc Natl Acad Sci U S A 2005 102 5074 5078 15809446 10.1073/pnas.0501526102 PMC555964

[B10] Dunlop RA Cox PA Banack SA Rodgers KJ The non-protein amino acid BMAA is misincorporated into human proteins in place of L-serine causing protein misfolding and aggregation PLoS One 2013 8 e75376 24086518 10.1371/journal.pone.0075376 PMC3783393

[B11] Rogers KJ Non-protein amino acids and neurodegeneration: the enemy within Exp Neurol 2014 253 192 196 24374297 10.1016/j.expneurol.2013.12.010

[B12] Caller TA Doolin JW Haney JF Murby AJ West KG Farrar HE Ball A Harris BT Stommel EW A cluster of amyotrophic lateral sclerosis in New Hampshire: a possible role for toxic cyanobacteria blooms Amyotroph Lateral Scler 2009 10 Suppl 2 101 108 19929741 10.3109/17482960903278485

[B13] Masseret E Banack S Boumédiène F Abadie E Brient L Pernet F Juntas-Morales R Pageot N Metcalf J Cox P Camu W Dietary BMAA exposure in an amyotrophic lateral sclerosis cluster from southern France PLoS One 2013 8 e83406 24349504 10.1371/journal.pone.0083406 PMC3862759

[B14] Marles RJ Barrett ML Barnes J Chavez ML Gardiner P Ko R Mahady GB Low Dog T Sarma ND Giancaspro GI Sharaf M Griffiths J United States pharmacopeia safety evaluation of spirulina Crit Rev Food Sci Nutr 2011 51 593 604 21793723 10.1080/10408391003721719

[B15] Scott PM Niedzwiadek B Rawn DF Lau BP Liquid chromatographic determination of the cyanobacterial toxin beta-n-methylamino-L-alanine in algae food supplements, freshwater fish, and bottled water J Food Prot 2009 72 1769 1773 19722418 10.4315/0362-028x-72.8.1769

[B16] Spacil Z Eriksson J Jonasson S Rasmussen U Ilag LL Bergman B Analytical protocol for identification of BMAA and DAB in biological samples Analyst 2010 135 127 132 20024192 10.1039/b921048b

[B17] Banack SA Metcalf JS Spacil Z Downing TG Downing S Long A Nunn PB Cox PA Distinguishing the cyanobacterial neurotoxin β-N-methylamino-L-alanine (BMAA) from other diamino acids Toxicon 2011 57 730 738 21329717 10.1016/j.toxicon.2011.02.005

[B18] Li A Fan H Ma F McCarron P Thomas K Tang X Quilliam MA Elucidation of matrix effects and performance of solid-phase extraction for LC-MS/MS analysis of β-N-methylamino-L-alanine (BMAA) and 2,4-diaminobutyric acid (DAB) neurotoxins in cyanobacteria Analyst 2012 137 1210 1219 22249403 10.1039/c2an15887f

[B19] Jiang L Aigret B De Borggraeve WM Spacil Z Ilag LL Selective LC-MS/MS method for the identification of BMAA from its isomers in biological samples Anal Bioanal Chem 2012 403 1719 1730 22526645 10.1007/s00216-012-5966-y

[B20] Jiang L Johnston E Åberg KM Nilsson U Ilag LL Strategy for quantifying trace levels of BMAA in cyanobacteria by LC/MS/MS Anal Bioanal Chem 2013 405 1283 1292 23180086 10.1007/s00216-012-6550-1

[B21] Glover WB Liberto CM McNeil WS Banack SA Shipley PR Murch SJ Reactivity of β-methylamino-L-alanine in complex sample matrixes complicating detection and quantification by mass spectrometry Anal Chem 2012 84 7946 7953 22905767 10.1021/ac301691r

[B22] Li A Tian Z Li J Yu R Banack SA Wang Z Detection of the neurotoxin BMAA within cyanobacteria isolated from freshwater in China Toxicon 2010 55 947 953 19822166 10.1016/j.toxicon.2009.09.023

[B23] Faassen EJ Presence of the neurotoxin BMAA in aquatic ecosystems: what do we really know? Toxins 2014 6 1109 1138 24662480 10.3390/toxins6031109 PMC3968380

[B24] Yoon JY Park HA Kang JH Kim KW Hur YI Park JJ Lee R Lee HH Prevalence of dietary supplement use in Korean children and adolescents: insights from Korea National Health and Nutrition Examination Survey 2007–2009 J Korean Med Sci 2012 27 512 517 22563216 10.3346/jkms.2012.27.5.512 PMC3342542

[B25] Kalafati M Jamurtas AZ Nikolaidis MG Paschalis V Theodorou AA Sakellariou GK Koutedakis Y Kouretas D Ergogenic and antioxidant effects of spirulina supplementation in humans Med Sci Sports Exerc 2010 42 142 151 20010119 10.1249/MSS.0b013e3181ac7a45

[B26] Snyder LR Cruz-Aguado R Sadilek M Galasko D Shaw CA Montine TJ Parkinson-dementia complex and development of a new stable isotope dilution assay for BMAA detection in tissue Toxicol Appl Pharmacol 2009 240 180 188 19716838 10.1016/j.taap.2009.06.025 PMC2753714

[B27] Hu Y Ziffer H Synthesis and optical resolution of the neurotoxin 2-amino-3-([^15^N]-methylamino) propanoic acid (BMAA) J Labelled Compounds Radiopharmaceuticals 1990 28 581 586

[B28] Yang Y Park Y Cassada DA Snow DD Rogers DG Lee J *In vitro* and *in vivo* safety assessment of edible blue-green algae, Nostoc commune var. sphaeroidesKützing and Spirulinaplantensis Food Chem Toxicol 2011 49 1560 1564 21473896 10.1016/j.fct.2011.03.052 PMC3107672

[B29] Banji D Banji OJ Pratusha NG Annamalai AR Investigation on the role of Spirulina platensis in ameliorating behavioural changes, thyroid dysfunction and oxidative stress in offspring of pregnant rats exposed to fluoride Food Chem 2013 140 321 331 23578649 10.1016/j.foodchem.2013.02.076

[B30] Hwang JH Chen JC Chan YC Effects of C-phycocyanin and Spirulina on salicylate-induced tinnitus, expression of NMDA receptor and inflammatory genes PLoS One 2013 8 e58215 23533584 10.1371/journal.pone.0058215 PMC3606192

[B31] Tobón-Velasco JC Palafox-Sánchez V Mendieta L García E Santamaría A Chamorro-Cevallos G Limón ID Antioxidant effect of Spirulina (Arthrospira) maxima in a neurotoxic model caused by 6-OHDA in the rat striatum J Neural Transm 2013 120 1179 1189 23430275 10.1007/s00702-013-0976-2

[B32] Gargouri M Ghorbel-Koubaa F Bonenfant-Magné M Magné C Dauvergne X Ksouri R Krichen Y Abdelly C El Feki A Spirulina or dandelion-enriched diet of mothers alleviates lead-induced damages in brain and cerebellum of newborn rats Food Chem Toxicol 2012 50 2303 2310 22504531 10.1016/j.fct.2012.04.003

[B33] Stromberg I Gemma C Vila J Bickford PC Blueberry- and spirulina-enriched diets enhance striatal dopamine recovery and induce a rapid, transient microglia activation after injury of the rat nigrostriatal dopamine system Exp Neurol 2005 196 298 307 16176814 10.1016/j.expneurol.2005.08.013

[B34] Escalona-Cardoso GN Paniagua-Castro N Pérez-Pastén R Chamorro-Cevallos G Spirulina (arthrospira) protects against valproic acid-induced neural tube defects in mice J Med Food 2012 15 1103 1108 23134463 10.1089/jmf.2012.0057

[B35] Pabon MM Jernberg JN Morganti J Contreras J Hudson CE Klein RL Bickford PC A spirulina-enhanced diet provides neuroprotection in an α-synuclein model of Parkinson’s disease PLoS One 2012 7 e45256 23028885 10.1371/journal.pone.0045256 PMC3445455

[B36] Abdel-Daim MM Abuzead SM Halawa SM Protective role of Spirulina platensis against acute deltamethrin-induced toxicity in rats PLoS One 2013 8 e72991 24039839 10.1371/journal.pone.0072991 PMC3767669

[B37] Downing S Banack SA Metcalf JS Cox PA Downing TG Nitrogen starvation of cyanobacteria results in the production of β-N-methylamino-L-alanine Toxicon 2011 58 187 194 21704054 10.1016/j.toxicon.2011.05.017

[B38] Holman BW Malau-Aduli AE Spirulina as a livestock supplement and animal feed J Anim Physiol Anim Nutr 2013 97 615 623 10.1111/j.1439-0396.2012.01328.x 22860698

